# 3D-Printed PLA/HA Composite Scaffolds: Balancing Mechanical Properties for Bone Tissue Engineering

**DOI:** 10.3390/ma19102083

**Published:** 2026-05-15

**Authors:** Muhamad Naseh Sajadi Budi, Muhammad Agus Kariem, Brilliant Dwinata, Yudi Mulyana Hidayat, Agung Budi Sutiono, Fathurachman Fathurachman, Wan Faisham Numan Wan Ismail, Yessicha Gracia Dwitama, Prapanca Nugraha

**Affiliations:** 1 Department of Orthopaedics, Faculty of Medicine, Universitas Padjadjaran, Bandung 40161, Indonesia; fathurachman@unpad.ac.id; 2 Department of Mechanical and Aerospace Engineering, Institut Teknologi Bandung, Bandung 40132, Indonesia; kariem@edc.ms.itb.ac.id; 3 Department of Mechanical Engineering, Universitas Jenderal Achmad Yani, Cimahi 40531, Indonesia; brilliant.dwinata@gmail.com; 4 Department of Obstetrics and Gynecology, Faculty of Medicine, Universitas Padjadjaran, Bandung 40161, Indonesia; yudi.mulyana@unpad.ac.id; 5 Department of Neurosurgery, Faculty of Medicine, Universitas Padjadjaran, Bandung 40161, Indonesia; a.b.sutiono@unpad.ac.id; 6 Department of Orthopaedics, Faculty of Medicine, Universiti Sains Malaysia, George Town 11800, Malaysia; wifaisham@gmail.com; 7 Department of Surgery, Faculty of Medicine, Universitas Padjadjaran, Bandung 40161, Indonesia; yessicha20001@mail.unpad.ac.id

**Keywords:** biomaterial, scaffold, polylactic acid, hydroxyapatite, 3D printing, fused deposition modeling

## Abstract

Bone tissue engineering requires biomimetic materials; however, pure polylactic acid (PLA) exhibits limited osteoinductivity and produces acidic byproducts upon degradation. To address these limitations, this study fabricated PLA scaffolds using fused-deposition modeling (FDM) with four distinct lattice structures (rectangular, triangular, gyroid, and 3D honeycomb) and incorporated hydroxyapatite (HA) at 0, 10, 20, and 30 wt% via injection molding. Mechanical properties were evaluated via compression, three-point bending, and tensile testing. The results revealed that increasing HA content significantly reduced structural strength and increased brittleness across all test modes. Specifically, specimens with 30 wt% HA exhibited a 70.8% reduction in bending strength relative to pure PLA (from 58.60 MPa to 17.07 MPa), while tensile strength decreased by 46.1% at just 10 wt% HA (from 37.54 MPa to 20.23 MPa). Although the triangular lattice achieved the highest absolute compressive load, the rectangular lattice provided a superior load-to-weight ratio and greater plastic deformation capacity before fracture. Consequently, these findings indicate that the rectangular pattern at 70% infill density combined with HA addition limited to ≤10 wt% represents the most mechanically balanced design for bone defect repair applications. Based on the mechanical characterization performed in this study, and drawing on published evidence regarding the biological properties of PLA/HA composites, these scaffolds represent a mechanically promising candidate for further evaluation in bone tissue regeneration. Biological validation through in vitro and in vivo studies is required before clinical relevance can be established.

## 1. Introduction

Bone defects are a frequent condition resulting from traumatic injuries, age-related degenerative diseases, tumors, or infections that require reconstruction [1,2]. More than 2.2 million bone graft procedures are performed worldwide annually, with the incidence rate expected to grow by 13% each year [3,4]. Bone graft procedures can be performed using autografts, allografts, and biomaterial-based bone graft substitutes [5]. Autologous bone is widely recognized as the gold standard for bone graft material, as it provides all three essential properties for bone regeneration: osteogenic, osteoconductive, and osteoinductive [4,6]. Despite these advantages, the use of autografts has significant drawbacks, including limited availability, secondary injuries, and donor-site morbidity, with an associated complication rate of 8–39% [7,8,9].

Bone tissue engineering is rapidly developing biomaterial-based alternatives to autografts, aiming to overcome the above-described limitations by leveraging advanced scaffolds, bioactive materials, and fabrication techniques [10,11,12]. Among synthetic polymers, polylactic acid (PLA) is currently used extensively in medical implants. As a favorable artificial polymer, most synthetic bone grafts use PLA as their base material due to its osteoconductive matrix, biocompatibility, and acceptability by bone tissue [13,14]. PLA has a medium degradation rate that is influenced by its molecular weight; the lower the molecular weight, the higher the degradation rate, which is suitable for matching the bone tissue growth rate [15]. PLA is widely used for 3D-printed bone scaffolds due to its processability; however, pure PLA exhibits low osteoinductivity and produces acidic degradation byproducts [16,17,18]. To overcome these drawbacks, combining PLA with bioactive ceramics such as hydroxyapatite (HA) has been reported to enhance osteoinductivity and buffer acidic degradation products; while HA at low concentrations and under optimized processing can improve composite modulus, the present study demonstrates that micron-scale HA addition progressively reduces strength at all concentrations tested, consistent with agglomeration-driven stress concentration effects documented in the literature [14,16].

Hydroxyapatite is extensively used as a bone substitute due to its high osteoconductivity and biocompatibility, closely mimicking the natural bone mineral [19,20]. HA is composed of calcium and phosphate ions, which are naturally occurring substances in the body [4]. No significant adverse local or systemic inflammatory responses have been reported resulting from the use of this material; thus, HA has been proven to be a safe bone substitute material [21]. While pure HA scaffolds are excellent for bioactivity and bone integration, their mechanical weakness restricts their use in load-bearing sites [22]. Composite strategies, especially those combining HA with polymers, are therefore essential to achieve the mechanical robustness required for clinical bone repair [23,24].

Incorporating bioactive ceramics like HA into PLA filaments enhances osteoconductivity and helps neutralize acidic PLA degradation byproducts, creating composite scaffolds with improved biological and structural properties [25,26]. PLA/HA composite scaffolds also show enhanced biocompatibility and bioactivity for bone tissue engineering without increasing inflammatory responses compared to controls [14,16]. Lattice structure, commonly known as infill pattern, is a critical design parameter for optimizing PLA scaffolds, enabling the fabrication of constructs that closely replicate natural bone while meeting both mechanical and regenerative requirements [27,28,29]. Consequently, PLA/HA scaffolds may be a promising candidate for clinical bone repair applications. There is significant variability in scaffold fabrication methods and HA ratios, making it difficult to determine optimal HA content for bone regeneration [17]. Across many studies, fused-deposition modeling (FDM) is consistently described as a promising technology for tissue engineering scaffolds because it enables solvent-free printing of diverse bioactive and biocompatible polymers into porous, patient-specific architectures with tunable mechanical properties, making it well suited for bone regeneration [30,31,32].

The present study systematically investigated 3D-printed PLA/HA composite scaffolds fabricated via FDM with varying HA ratios and four infill patterns (rectangular, triangular, gyroid, and 3D honeycomb). The scaffolds were further characterized by performing compressive, bending, and tensile tests to identify mechanically balanced designs suitable for bone tissue engineering. Specifically, the present study addresses the gap in the literature by providing a simultaneous, multi-modal mechanical characterization—compression, three-point bending, and tensile—across four FDM infill geometries and four HA loading levels, enabling a systematic, data-driven identification of the mechanically balanced scaffold design for large bone defect repair.

## 2. Materials and Methods

### 2.1. Materials

The materials used in this study included PLA as the polymer matrix and HA as the bioactive filler. A commercially available raw PLA in the form of pellets with a nominal diameter of approximately 1 mm was used as the feedstock material. All specimens were fabricated under controlled and identical processing conditions to ensure repeatability and to minimize variability associated with the material and manufacturing process.

Porous PLA scaffolds were fabricated via FDM-based 3D printing technology that extrudes thermoplastic material layer by layer through a heated nozzle. HA contents of 0, 10, 20, and 30 wt% were used together with an unmodified PLA control (0% HA). These four groups are hereafter referred to as the control and the HA-reinforced series, respectively. Due to cost and supply limitation in Indonesia, the HA used was conventional (micron-scale) hydroxyapatite rather than nano-hydroxyapatite (n-HA), which limited filament uniformity but still provided osteoconductive sites and acid-buffering capability.

### 2.2. Scaffold Design

Lattice structures are classified based on their underlying architectural topology, typically into two principal categories: strut-based and surface-based lattice structures [33]. Each lattice structure exhibits fundamentally different mechanical behavior, thereby determining their suitability for specific application scenarios.

When designing a 3D-printed component, the designer has several options: completely filling the part, which requires substantial time and material and thus increases costs but offers enhanced mechanical strength, or utilizing infill patterns to mimic certain mechanical properties and ensure stability while reducing weight. Prior additive manufacturing (AM) studies consistently support porous, highly interconnected scaffolds that mimic bone’s internal structure [22,31].

**Figure 1:**
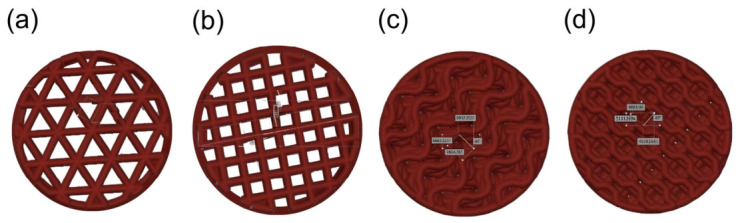
AutoCAD images of the scaffold’s infill patterns. From left to right: (**a**) Triangular. (**b**) Rectangular. (**c**) Gyroid. (**d**) 3D honeycomb.

The present study restricted the analysis to four infill patterns that are available on PrusaSlicer that was used in this study, namely Prusa i3 MK3S+ 3D printer, selected to yield a target scaffold porosity of 30%. Four lattice geometries were generated in PrusaSlicer: (1) rectangular pattern, (2) triangular pattern, (3) gyroid pattern, and (4) 3D honeycomb pattern. Based on a prior study examining the mechanical behavior of fourteen common infill patterns under compressive testing, the two highest-ranking patterns based on strength-to-weight ratio—rectangular and triangular—were selected [34]. The other two patterns selected were gyroid and 3D honeycomb, both of which have been reported to exhibit high mechanical strength [35]. The 3D honeycomb infill pattern is optimal for maximizing compressive and tensile strength in bone scaffolds, while the gyroid pattern provides exceptional resistance to shear and complex loading, along with high fatigue durability and biological compatibility [36,37,38,39]. The scaffold model was designed using AutoCAD 2023 (Autodesk) before being processed in PrusaSlicer for fabrication using a Prusa i3 MK3S+ printer. Figure 1 illustrates the AutoCAD 2023 (Autodesk) images of each pattern as a visual reference in the design process. These models were defined to ensure that the intended geometrical variations were accurately reflected in the scaffold architecture prior to final modeling.

Previous studies have demonstrated that macropore sizes of 100–500 µm in porous scaffolds are capable of effectively facilitating osteogenic cell migration, ensuring adequate fluid transport and vascularization [40,41,42]. The shape of each pore follows the underlying infill pattern: rectangular grids produce straight-line channels, triangular infill creates intersecting triangular voids, gyroid forms a continuous triply periodic minimal surface (TPMS), and honeycomb yields stacked hexagonal cells.

The macropore size of 700–1200 µm fabricated in this study substantially exceeds the recommended 300–500 µm range for osteogenic cell migration and vascularization [40,41]. This deviation is a constraint of the FDM process parameters employed—specifically the 0.4 mm nozzle diameter, 0.3 mm layer height, and 60 mm/s print speed—which limit the minimum achievable pore feature size. Future studies should explore finer nozzle diameters or optimized slicing parameters to reduce macropore size toward the biologically recommended range. The larger pore size observed here may limit initial cell seeding efficiency and early vascularization, representing an acknowledged biological limitation of the present scaffold design.

**Figure 2:**
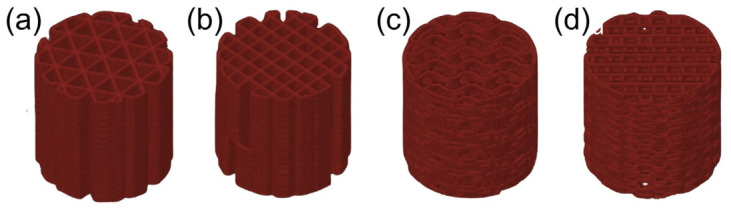
SolidWorks images of three-dimensional computational models. From left to right: (**a**) Triangular. (**b**) Rectangular. (**c**) Gyroid. (**d**) 3D honeycomb.

A computational cylinder-shaped porous scaffolds with both the diameter and height of 10 mm were designed using SolidWorks software as presented in Figure 2. The scaffold 3D models were designed using SolidWorks 2022 prior to STL file export and slicing in Prusa i3 MK3S+ 3D printer. The printing path of the 3D models was exported as a G-code file using PrusaSlicer software with built-in selection of internal structure and infill density of the design. The printing parameters were set as follows: a layer height of 0.2 mm, a nozzle speed of 60 mm/s, and the perimeter was set to 0 because the aim was to measure the mechanical properties of the internal structure. The 3D-printed scaffolds were then fabricated by transferring the G-code file to the Prusa i3 MK3S+ machine.

### 2.3. Fabrication of Pure PLA Scaffolds

The pure PLA scaffold specimens were fabricated using an Original Prusa i3 MK3S+ 3D printer (Prusa Research, Prague, Czech Republic). The original Prusa i3 MK3S+ machine uses a heated nozzle to extrude thermoplastic material layer by layer. To achieve comparable porosity, rectangular and triangular patterns were printed at 70% infill density, while gyroid and 3D honeycomb were printed at 40% infill density. This differentiation arises from intrinsic geometric differences, as grid and triangular patterns exhibit higher inherent porosity than gyroid and honeycomb architectures at equivalent density settings [43,44]. Density parameters were adjusted to account for these structural disparities, ensuring comparable porosity while maintaining compatibility with slicing constraints and facilitating potential bone ingrowth [44,45]. The quality of FDM-printed objects is significantly influenced by printing parameters affecting both mechanical performance and dimensional accuracy. All the mechanical testing specimens were printed under the fixed parameters for FDM printing summarized in Table 1.

**Figure 3:**
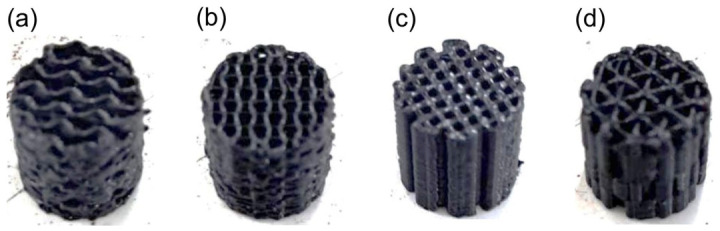
Digital photographs of pure PLA 3D printed scaffolds. From left to right: (**a**) Gyroid. (**b**) 3D honeycomb. (**c**) Rectangular. (**d**) Triangular.

The pure PLA internal-structure groups use five specimens per geometry, and the PLA/HA groups use three specimens per ratio. The digital photographs of pure PLA 3D printed scaffolds with four different lattice structures are presented in Figure 3. The four geometries were gyroid, rectangular, triangular, and 3D honeycomb, and each internal structure was printed as five pure PLA-based specimens for the compressive test. For the compressive test, each of those same four geometries was also printed as five specimens. The total number was 10 specimens per geometry overall, 20 specimens per test type, and 40 printed specimens in total across the two geometry-based tests of pure PLA scaffolds. For each HA-reinforced composition, three specimens were produced by injection molding per test type, yielding a further 36 injection-molded specimens.

### 2.4. Fabrication of PLA/HA Scaffolds

**Figure 4:**
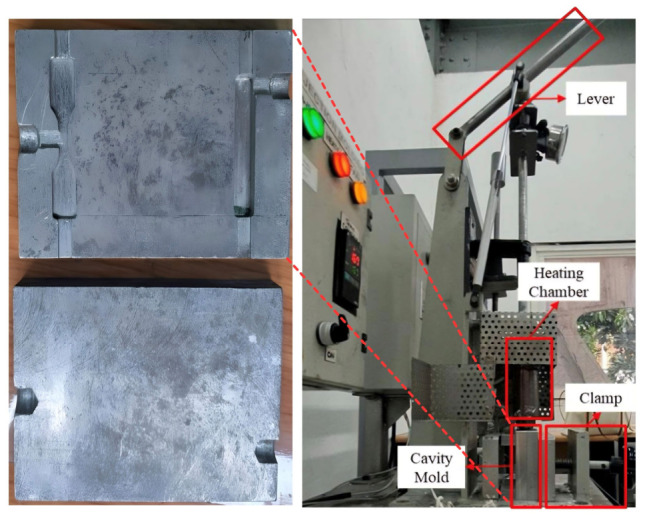
Schematic diagram of the injection molding process.

Specimen preparation was performed via injection molding using a custom-built manual injection molding machine at the Polymer-Composite Laboratory, Institut Teknologi Bandung (Bandung, Indonesia). Prior to injection, the PLA/HA compound was manually loaded into the heating chamber. Micron-scale HA powder was blended with neat PLA at the ratio of HA-reinforced series. Because the PLA/HA scaffold could not be extruded reliably due to nozzle clogging, the test specimens were produced using a manually operated injection molding machine. PLA pre-mixed with specified HA ratios was heated to 165–170 °C in a chamber, held for 1–2 min, and then cooled before machining to final dimensions. Once the material was fully melted, the mold was secured within the clamp, and the molten PLA/HA mixture was rapidly injected into the aluminum cavity molds by actuating the lever, as illustrated in Figure 4. The cavity mold was fabricated by PT Kawani Tekno Nusantara (Bandung, Indonesia). The lever was actuated manually over approximately 2–3 s to inject the molten material. The mold was held under pressure for approximately 30 s before release to minimize flash and shrinkage voids.

Mechanical testing specimens were prepared in compliance with the appropriate standards, meeting the shape, dimensional, and test machine requirements for each specimen type. Compressive properties were evaluated in accordance with ISO 604:2002 [46] on cylindrical specimens (diameter and height: 10 ± 2 mm) at a crosshead speed of 1 mm s^−1^ to record load–displacement curves and determine the ultimate compressive load and deformation behavior for each infill pattern and HA content. Flexural properties were evaluated according to ASTM D790-17 [47] using a three-point bending fixture with a 51.2 mm support span; the test measured load versus deflection to obtain ultimate bending strength and load-to-weight ratios for the four lattice designs. Flexural testing was conducted using a TENSILON RTF-1310 universal testing machine (A&D Company, Ltd., Tokyo, Japan) with a maximum force capacity of 10 kN equipped with a three-point bending fixture. Due to machine limitations and time constraints, the bending test specimen geometry was determined as follows: 13 mm for thickness, 25 mm for width, 78 mm for outer span, and 117 mm for specimen length. The tensile properties were assessed in accordance with ASTM D638-14 [48] on Type V dog-bone specimens at a crosshead speed of 1 mm min^−1^, providing ultimate tensile strength and ductility data for both pure PLA and PLA/HA composites.

**Figure 5:**
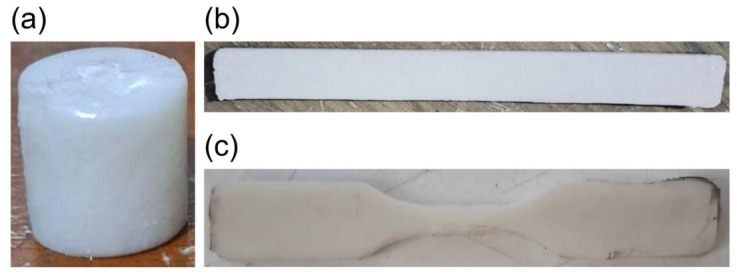
Fabricated PLA/HA composite scaffolds. (**a**) Compressive tests’ specimen. (**b**) Bending tests’ specimen. (**c**) Tensile tests’ specimen.

For FDM 3D-printed specimens, surface finishing or machining was unnecessary, as no support structures were required and the surfaces intended for compression or bending testing were sufficiently smooth. However, injection-molded specimens retained excess material after removal from the mold, which required machining to achieve clean surfaces. Bench grinders and Struers grinding machines were used to machine the specimen surfaces. Surface grinding was performed using Struers abrasive paper in a sequence of P120, P240, and P400 grit to achieve a smooth, flat test surface. Bench grinding was used only to remove gross excess material prior to the Struers finishing sequence. 3D-printed scaffolds are presented in Figure 5.

### 2.5. Determination of Mechanical Properties

To evaluate the mechanical properties of the 3D-printed PLA/HA scaffolds, two experimental series were conducted. The first series investigated the influence of internal architecture by fabricating pure PLA scaffolds with rectangular, triangular, gyroid, and 3D honeycomb infill patterns (70% density for rectangular/triangular; 40% density for gyroid/honeycomb) and comparing their compressive, bending, and tensile responses. Tests were conducted between unlubricated steel compression platens. No end-constraint or anti-barreling fixture was used, consistent with ISO 604:2002 [46] guidance for polymer specimens. The crosshead displacement rate for the three-point bending test was set to 2 mm min^−1^, in accordance with ASTM D790-17 [47] for the specimen geometry used.

It is important to note that the four infill patterns were not compared at identical infill densities: rectangular and triangular patterns were printed at 70% density, whereas gyroid and 3D honeycomb were printed at 40% density. This difference was intentional—to achieve comparable overall scaffold porosity (approximately 30%) across all four geometries, given that each pattern generates a different internal architecture at the same nominal density. As a consequence, the observed mechanical differences between groups reflect the combined effect of both geometry and infill density, and conclusions about geometric superiority in isolation cannot be drawn from the present study. When assessed at equivalent densities, published literature indicates that honeycomb exhibits the highest compressive strength, followed by gyroid, rectangular, and triangular patterns [49,50], which is consistent with our data once the density differential is considered. Future studies should compare all four geometries at identical infill densities to decouple the geometric and density contributions to mechanical performance.

The second series examined the effect of HA reinforcement by producing solid specimens through injection molding containing 0%, 10%, 20%, and 30% wt% HA. Each batch was subjected to the same three mechanical tests to evaluate how increasing the HA ratio altered strength, stiffness, and brittleness. In vitro studies consistently show that HA at low concentrations (0–5%) does not inhibit cell proliferation, while higher HA content can significantly alter scaffold mechanics and printability [51,52]. Low HA loadings (around 0–10%) in printable composites consistently support cell attachment and proliferation with no meaningful cytotoxicity, including for fibroblasts, chondrocytes, and osteogenic cells [53,54,55]. As HA content rises toward and above ~30%, materials generally become stiffer, more brittle, and harder to print with high shape fidelity, which can limit scaffold performance [53,56]. Crosslinking and polymer blending can help optimize these properties for specific applications [57]. Therefore, this study limits the selected HA ratios to 0%, 10%, 20%, and 30% to be incorporated with PLA scaffolds. These experimental procedures together identified the mechanically optimal scaffold geometry and HA content for load-bearing bone defect repair applications.

### 2.6. Stress–Strain Curve Determination

The compressive stress–strain characteristics were obtained from compressive load–displacement measurements acquired during uniaxial compression testing. Engineering stress (σ) was determined by dividing the applied load (F) by the initial cross-sectional area (A) of the specimen, expressed as σ = F/A, with load values converted from kN to N to maintain unit consistency (MPa = N/mm^2^). Engineering strain (ε) was calculated as ε = ΔL/L0, where ΔL represents the axial displacement and L0 is the initial specimen height. The analysis was conducted under the assumption of homogeneous deformation and constant cross-sectional area. These stress–strain relationships were used to characterize the mechanical response of the lattice structures.

### 2.7. Statistical Analysis

Five specimens per HA ratio group were used for compressive testing, whereas three specimens per HA ratio group were used for bending and tensile testing. Mechanical properties are presented as mean ± standard deviation (SD) in MPa. Between-group differences were evaluated using the Kruskal–Wallis test, followed by Dunn’s post hoc test with Holm multiplicity correction for pairwise comparisons. A *p*-value of less than 0.05 was considered statistically significant.

## 3. Results and Discussion

### 3.1. Fabrication and Characterization of PLA/HA Composite Scaffolds

Unlike soft tissues, bone fractures heal through a regenerative process involving the formation of new bone rather than the development of fibrotic tissue [58,59]. As the gold standard for bone substitution, autografts have limited tissue supply, cause donor site morbidity, and involve complex operative procedures [8]. Allografts, on the other hand, carry the risk of immune rejection and disease transmission [60]. Due to the notable disadvantages of both autografts and allografts, numerous synthetic bone substitutes are being investigated as emerging materials for bone grafting. Although PLA is considered an appropriate material due to its good osteoconductivity and degradation profile, it still produces acidic byproducts upon degradation [61]. To address these well-known limitations, HA has been incorporated into scaffolds to improve the degradation rate and stimulate osteogenesis [6]. However, pure HA scaffolds possess insufficient intrinsic mechanical strength for the reconstruction of large or load-bearing bone defects [62]. Moreover, increasing HA content progressively alters the microstructure of the PLA matrix, which directly affects scaffold mechanical performance—a key finding supported by the present experimental results.

The ideal scaffold for bone tissue engineering should exhibit superior biocompatibility, controlled biodegradability, and adequate mechanical integrity to promote effective bone regeneration [63,64]. Numerous biopolymers have been investigated for scaffold fabrication with the goal of developing a most mechanically balanced bone graft material. For instance, PLA/HA composite scaffolds have demonstrated considerable potential for tissue engineering and restoring maxillofacial defects [65]. Synthetic HA has been shown to support new bone formation, osteogenic marker expression and matrix mineralization [66]. While PLA is recognized for its excellent biocompatibility and biodegradability, it still has relatively low mechanical strength and osteoconductivity [61]. The present study sought to characterize the influence of varying HA contents and infill patterns on scaffold porosity and mechanical performance. Accordingly, this study combined PLA with the HA-reinforced series and fabricated the 3D printed scaffolds with the objective of identifying scaffold designs that balance mechanical integrity for large bone defect repair.

Prior studies have demonstrated that integrating bioactive ceramics with biodegradable polymers—as in PLA/HA composites—effectively balances bioactivity and mechanical integrity within the scaffold [61]. Uniform distribution of HA particles on the scaffold surface provides abundant bioactive sites conducive to bone regeneration. Furthermore, the degradation rate of PLA/HA scaffolds is positively correlated with HA content, because HA accelerates water uptake and increases scaffold porosity, thereby facilitating more rapid replacement of the scaffold by newly formed bone tissue [13].

Hydroxyapatite improves the bioactivity of PLA-based scaffolds by providing osteoconductive sites that favor cell attachment and by neutralizing the acidic byproducts released during PLA degradation, thereby helping to maintain a more favorable local pH for bone formation [25]. Notably, the acceleration of scaffold degradation observed with increasing HA content is not attributable to greater intrinsic biodegradability of HA relative to PLA; rather, it results from HA-induced increases in porosity and water uptake, combined with the acid-buffering effect of HA on the degradation microenvironment.

### 3.2. Mechanical Test Results of Pure PLA Scaffolds

#### 3.2.1. Stress Localization at Lattice Junction Regions

The mechanical behavior of lattice structures is strongly governed by the local stress state at junction regions—the nodes where individual struts, walls, or surfaces converge. These regions are well-established sites of stress concentration and fatigue crack initiation, and junction geometry has been shown to be a primary determinant of stress concentration severity in periodic lattice structures [67]. In the present study, post-failure examination of fractured specimens revealed that fracture surfaces consistently initiated at or near junction regions for the rectangular and triangular patterns. Orthogonal node junctions in rectangular pattern create 90° re-entrant corners that act as sharp stress concentrators under both compressive and bending loading [68]. Acute-angle junctions in triangular pattern generate higher local stress amplification than rectangular nodes under shear loading, but the triangulated load path means that the junction is loaded primarily in tension and compression rather than bending, which partially mitigates the stress concentration [69]. In additively manufactured cubic-type lattices, stress concentration at nodes is identified as a key cause of early damage and poor scale-up performance [70]. Adding fillets, minimal surfaces, or plates redistributes stresses, reduces concentration at nodes, and improves fatigue or compressive performance [71]. These observations are consistent with published findings that sharp-cornered junctions in rectangular-cell and triangular-cell lattices exhibit substantially elevated stress concentration relative to smooth junction geometries [67].

TPMSs are very smooth, without sharp edges or node-like connection points, in contrast to conventional strut lattices [72]. For the gyroid pattern, fracture propagated along the minimal surface without localized junction failure, reflecting the smooth, continuous shells, and fully interconnected pores of TPMS topology, which mitigates stress concentrations and minimizes curvature discontinuities that typically initiate premature failure in sharp-cornered lattice architectures [73]. A study using FE analysis shows that under out-of-plane compression, hexagonal and related honeycomb patterns develop localized buckling in cell walls that then evolves into progressive folding and crushing, giving rise to a gradual, often plateau-like load–displacement response rather than a single sudden fracture event [74]. A quantitative assessment of stress concentration factors for each junction geometry via FE analysis is identified as a priority for future work and would allow the experimental failure modes observed here to be interpreted at a local, mechanistic level.

#### 3.2.2. Compressive Test Results

**Figure 6:**
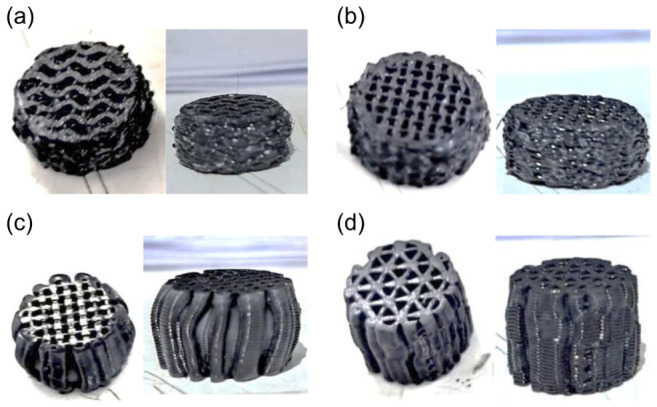
Representative photographs of the four PLA scaffold types after compressive test. (**a**) Gyroid. (**b**) 3D honeycomb. (**c**) Rectangular. (**d**) Triangular.

Compressive tests were conducted on pure PLA scaffolds with varying infill patterns to characterize the mechanical behavior of all four specimen types. Under compressive loading, the gyroid specimens retained overall structural integrity without catastrophic failure; however, visible barreling deformation was observed, as illustrated in Figure 6. This deformation is attributed to interfacial friction between the specimen and the loading platens, which constrains lateral expansion at the contact surfaces and promotes non-uniform stress distribution along the specimen height. The occurrence of barreling indicates that the customized internal architecture of the gyroid scaffold introduces localized structural inhomogeneities, resulting in uneven stress distribution and localized instability rather than uniform load-bearing behavior across the specimen.

**Figure 7:**
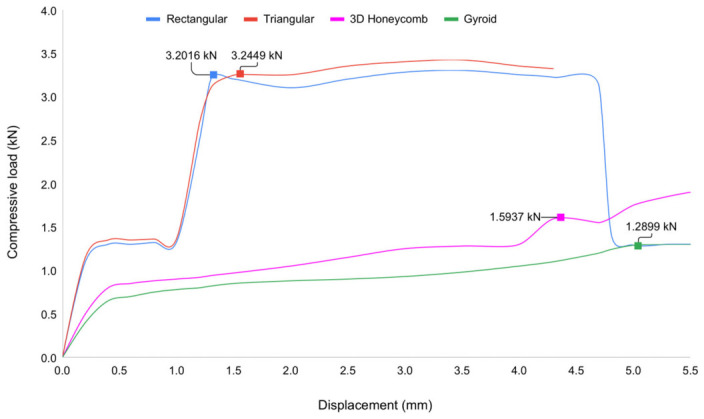
Comparison of the compressive load and displacement curves.

The triangular scaffold exhibited the highest ultimate compressive load (3.24 kN), followed closely by the rectangular scaffold (3.20 kN); in contrast, the gyroid and 3D honeycomb scaffolds carried substantially lower compressive loads (1.29 kN and 1.59 kN, respectively). Under three-point bending, the rectangular and triangular architectures again achieved the greatest ultimate loads (approximately 406 N and 412 N, respectively) and the highest specific load-to-weight performance, whereas the gyroid and 3D honeycomb patterns showed lower peak bending load but larger deformation prior to failure, consistent with greater ductility. Values represent means of five specimens per group (*n* = 5); numerical mean ± SD data are provided in the text. These load-based comparisons pertain specifically to the porous pure PLA scaffold architectures as shown in Figure 7.

**Figure 8:**
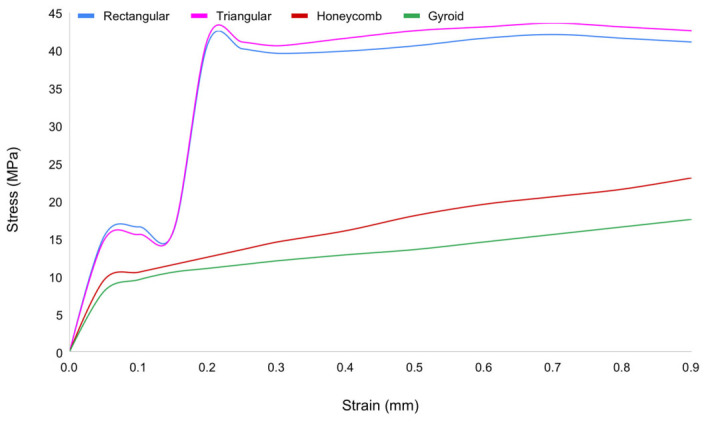
Compressive stress–strain curves of lattice structures with different architectural configurations (rectangular, triangular, 3D honeycomb, and gyroid).

The compressive stress–strain curves demonstrate a clear dependence of mechanical performance on lattice structure as shown in Figure 8. Among the four infill architectures investigated, the triangular structure exhibited the highest compressive strength at 41.32 MPa, followed by the rectangular structure at 40.76 MPa. Both geometries displayed a well-defined elastic–yield–plateau–densification sequence, with a distinct plateau region indicative of progressive structural collapse governed by strut buckling and nodal junction failure. The 3D honeycomb structure demonstrated intermediate mechanical performance, with a peak compressive stress of 20.29 MPa and a more gradual transition into the plateau region, suggesting a concurrent contribution of localized deformation and distributed load transfer mechanisms. The gyroid structure, representing a TPMS-based architecture, recorded the lowest peak compressive stress at 16.42 MPa; however, it exhibited a smooth and continuous stress–strain response in the absence of a pronounced yield point, which is characteristic of the progressive and uniform deformation behavior inherent to minimal surface geometries. This behavior reflects its continuous surface morphology and absence of discrete nodes, which facilitate uniform stress distribution and suppress localized failure. Overall, strut-based architectures favor higher load-bearing capacity, whereas TPMS-based structures provide enhanced deformation stability and progressive energy absorption.

**Figure 9:**
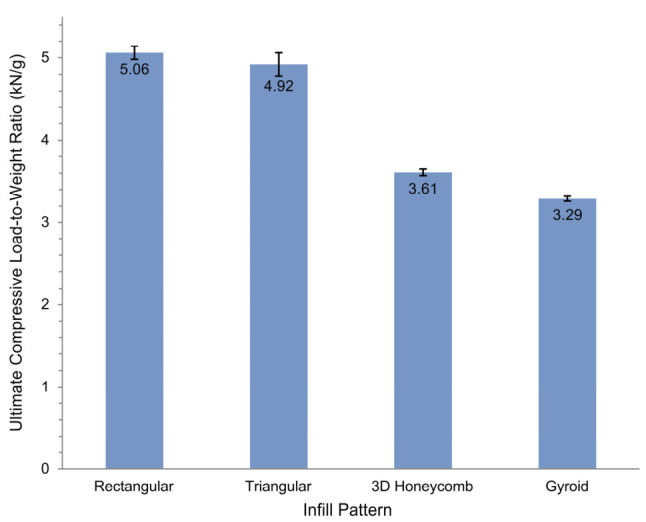
Ultimate compressive load-to-weight ratio of 3D-printed pure PLA specimens. Error bars represent standard deviation (*n* = 5).

As shown in Figure 9, the rectangular pattern exhibited the highest ultimate compressive load-per-weight ratio, followed by the triangular, 3D honeycomb, and gyroid patterns. While the gyroid pattern exhibited the highest fracture toughness—which is advantageous for bone scaffolds where brittle fracture could pose a risk of patient injury—it also demonstrated the lowest maximum bending load among all patterns tested. Given that a bone scaffold functions primarily as a temporary support structure, excessive deformation is functionally equivalent to failure even in the absence of fracture. Therefore, the rectangular pattern is more suitable than the triangular pattern as the internal scaffold structure, offering superior load-to-weight efficiency alongside meaningful plastic deformation capacity before failure.

#### 3.2.3. Bending Test Results

**Figure 10:**
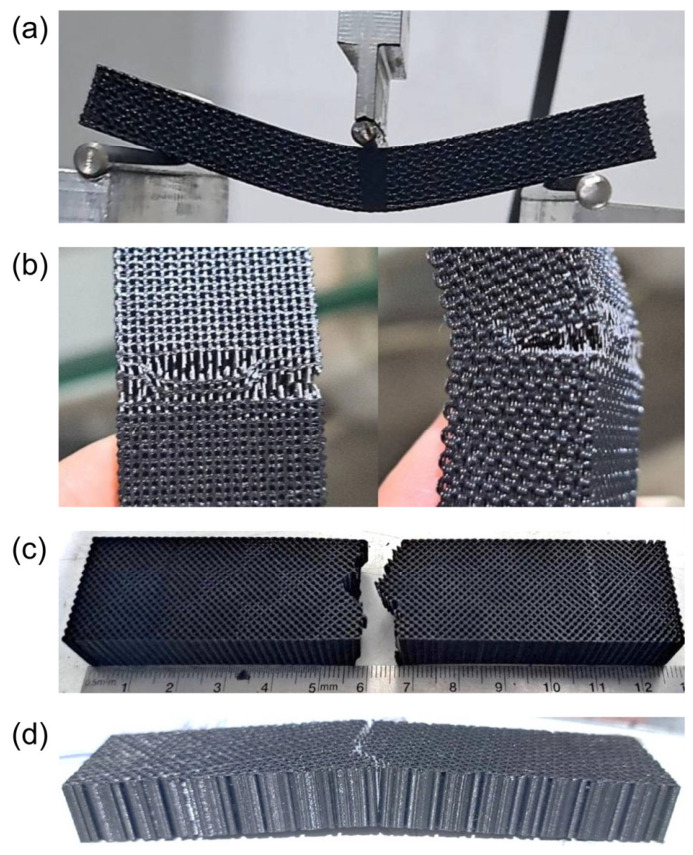
Representative photographs of the four PLA scaffold types after bending test. (**a**) Gyroid pattern. (**b**) 3D honeycomb pattern. (**c**) Rectangular pattern. (**d**) Triangular pattern.

The three-point flexural test was performed to evaluate scaffold behavior under bending moments, which simulate the combined tensile and compressive stresses that a bone implant experiences in vivo. This test provides the flexural strength (maximum load before fracture), the flexural modulus (stiffness during bending), and information on the elastic–plastic transition and the extent of plastic deformation before failure. As illustrated in Figure 10, the post-failure morphology of each scaffold varied distinctly according to its infill architecture. The gyroid specimen (a) exhibited large-scale, progressive flexural deformation without a clearly defined single fracture plane, consistent with the smooth, node-free topology of triply periodic minimal surfaces that distributes stress continuously across the structure and suppresses localized crack initiation. The 3D honeycomb specimen (b) similarly underwent notable plastic deformation prior to failure; however, visible cell-wall buckling and partial delamination along the stacking direction were evident, reflecting the progressive, layer-by-layer folding mechanism characteristic of hexagonal cellular architectures under out-of-plane bending. In contrast, the rectangular specimen (c) displayed a discrete, well-defined fracture line oriented perpendicular to the loading axis, indicative of abrupt tensile failure at the outermost fiber once the ultimate bending load was reached, with comparatively limited plastic deformation beyond the fracture point. The triangular specimen (d) exhibited a similar sharp, brittle-like fracture pattern; however, the fracture surface appeared more irregular, consistent with the higher local stress amplification at the acute-angle nodal junctions under flexural loading, which promotes crack propagation along multiple strut interfaces rather than a single dominant plane.

**Figure 11:**
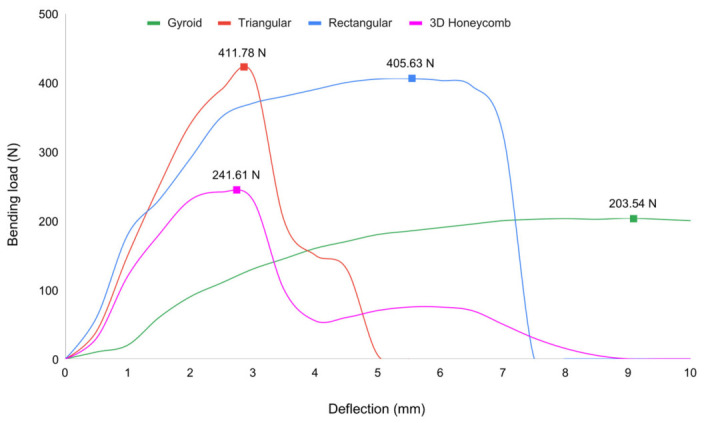
Comparison of bending load and deflection rates of four types of internal structure.

The bending test results are shown in Figure 11. Pure PLA scaffolds with rectangular and triangular infill patterns (both printed at 70% infill density) carried the greatest loads, while those with gyroid and 3D honeycomb patterns (printed at 40% density) were substantially weaker.

**Figure 12:**
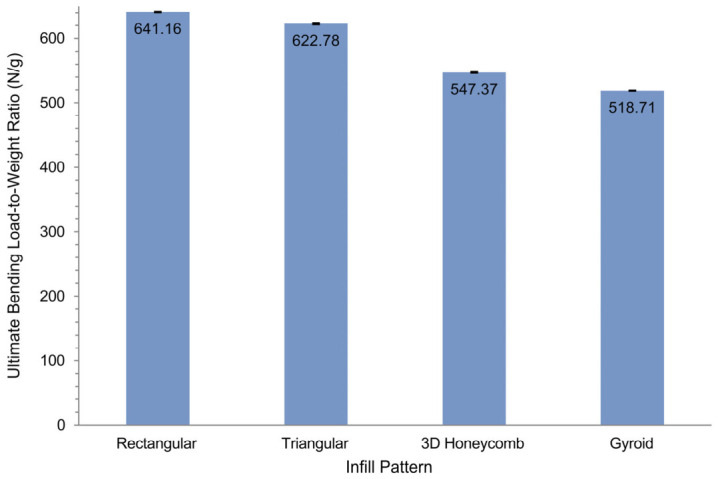
Ultimate bending load-to-weight ratio of 3D-printed pure PLA specimens. Error bars represent standard deviation (*n* = 3).

The rectangular and triangular pure PLA porous scaffold designs reached the highest ultimate bending loads, with the rectangular specimen sustaining approximately 406 N (≈11.3 MPa) and the triangular specimen approximately 412 N (≈11.5 MPa). In contrast, the gyroid scaffold sustained approximately 205 N (≈5.7 MPa) and the 3D honeycomb approximately 243 N (≈6.7 MPa). The load–displacement curves show that the gyroid and 3D honeycomb scaffolds deformed over a larger displacement range before failure, indicating higher ductility but lower peak strength, whereas the rectangular and triangular scaffolds fractured at lower deflection (~5–6 mm) with a sharp load drop after reaching the ultimate load. All four patterns displayed a clear elastic region followed by a short plateau, consistent with typical porous PLA mechanical behavior. The rectangular infill pattern yielded the highest ultimate bending load-to-weight ratio at 641.16 N/g, followed by triangular (622.78 N/g), 3D honeycomb (547.37 N/g), and gyroid (518.71 N/g) as shown in Figure 12. The difference between the rectangular and triangular patterns was 18.38 N/g (2.9%), while the rectangular pattern exceeded the gyroid by 122.45 N/g (23.6%). Standard deviations were small across all groups, indicating high inter-specimen consistency in both fabrication and testing. Importantly, these results refer specifically to the bending response of porous pure PLA scaffolds with different internal architectures (Figure 11 and Figure 12) and should not be compared directly with the solid PLA/HA composite coupon properties reported in Table 2, which represent a different specimen type and testing set.

These findings are consistent with prior research on 3D-printed parts with various infill patterns, in which rectangular and triangular infills were reported to exhibit lower compressive strength than several other patterns when compared at equivalent infill densities, owing to their lower material connectivity [75]. However, the results of this study show that rectangular and triangular infills outperformed gyroid and 3D honeycomb patterns in both compressive and bending tests. This discrepancy arises from the difference in infill densities used in this experimental study: rectangular and triangular patterns were printed at 70% infill density, while gyroid and honeycomb patterns were printed at only 40% infill density to achieve comparable scaffold porosity. When compared at equivalent infill density, the honeycomb pattern has been reported to exhibit the highest compressive strength, followed by gyroid, rectangular, and triangular patterns [76,77]. The rectangular pattern at 70% infill density can withstand high bending loads while simultaneously exhibiting a greater capacity for plastic deformation compared to the other patterns evaluated at their respective densities.

### 3.3. Effects of HA on Mechanical Properties of PLA Scaffolds

All HA-containing specimens were fabricated by manual injection molding, as repeated attempts to extrude a PLA/HA filament for FDM failed due to nozzle clogging; consequently, all 3D-printed samples consisted of pure PLA only. HA addition did not result in a uniform particle distribution. Higher HA ratios produced noticeable clumping, voids, and surface roughness in the molded parts. Mechanically, increasing HA content reduced strength across all tests: compressive, bending, and tensile capacities all declined progressively. Furthermore, the material became markedly more brittle, with a 50% loss in tensile strength observed at 20% and 30% HA ratios [14]. As a result, the HA-to-PLA ratio must be carefully optimized to balance mechanical integrity and bioactivity in bone scaffolds.

It should be noted that the FDM-fabricated scaffolds (pure PLA, varying lattice geometry) and the injection-molded specimens (solid PLA/HA composites, varying HA content) were produced by different manufacturing routes, which constitutes an acknowledged methodological limitation. FDM and injection molding differ in thermal history, cooling rate, degree of crystallinity, and internal void distribution, all of which influence mechanical behavior independently of composition. Consequently, direct cross-method comparisons of absolute mechanical values must be interpreted with caution. To isolate the effect of HA content, comparisons within the injection-molded series (0%, 10%, 20%, 30% HA) are the most valid, as these specimens share the same fabrication method. The decision to use injection molding for HA-containing specimens was driven by the repeated failure of FDM extrusion due to nozzle clogging from micron-scale HA particles—a limitation that has been documented in the literature for conventional HA particle sizes. Future work should prioritize the development of printable PLA/HA filaments using nano-HA or surface-treated HA to enable a fully FDM-based comparative study.

A further limitation of this study is the absence of microstructural characterization, including scanning electron microscopy (SEM), HA particle dispersion analysis, and quantitative porosity measurement (e.g., via micro-CT). The observed progressive reduction in mechanical strength with increasing HA content is consistent with published SEM evidence of HA particle agglomeration and associated void formation at HA loadings above 10 wt%, which serve as stress concentrators under mechanical loading. However, without direct microstructural evidence from the present specimens, the precise mechanisms underlying the mechanical trends observed here cannot be definitively confirmed. Future studies should incorporate SEM and micro-CT analysis to establish structure–property relationships more rigorously.

This section reports the mechanical properties of PLA/HA composite specimens fabricated by injection molding. Two types of comparison are made: (1) within the injection-molded series—comparing 0% HA (control), 10%, 20%, and 30% HA specimens to evaluate the effect of HA content under a consistent manufacturing method; and (2) between the injection-molded 0% HA control and the FDM-printed pure PLA scaffold—to assess the effect of manufacturing method on mechanical properties in the absence of HA. These two comparisons are reported and discussed separately. Readers should note that comparisons across fabrication methods are exploratory and subject to the confounding effect of microstructural differences arising from the different manufacturing processes, acknowledged as a key limitation of this study.

Several studies have demonstrated that adding HA as an additive to PLA for bone scaffolds enhances the scaffold’s biological activity and can accelerate its degradation rate. However, with respect to mechanical properties, adding HA directly alters the microstructure of the PLA matrix, which in turn affects the scaffold’s structural matrix and is directly reflected in its mechanical performance. As a limitation, this study does not address aspects beyond the mechanical properties and theoretical bioactivity of the scaffolds, such as in vitro and in vivo experiments to evaluate biocompatibility, cytotoxicity, antibacterial performance, osteogenesis induction, and other biological properties, which require further work in the future. In addition to mechanical testing, future studies may also examine the hardness values of the scaffolds.

#### 3.3.1. Compressive Testing

After compressive loading, the deformed specimens exhibited barreling even though the FDM printer was set to 100% infill density with a rectilinear pattern—which should theoretically produce solid specimens. This barreling in the 0% HA specimens indicates that voids were still present. This is because 100% infill density refers to the percentage of the part filled with printed material, not the proportion of the printed material that is fully solid. Consequently, voids can still arise due to the printing process itself or from printing parameters that generate small gaps. These gaps cause localized instability, as the specimen is unable to withstand the applied load uniformly, causing the surface to bulge.

**Figure 13:**
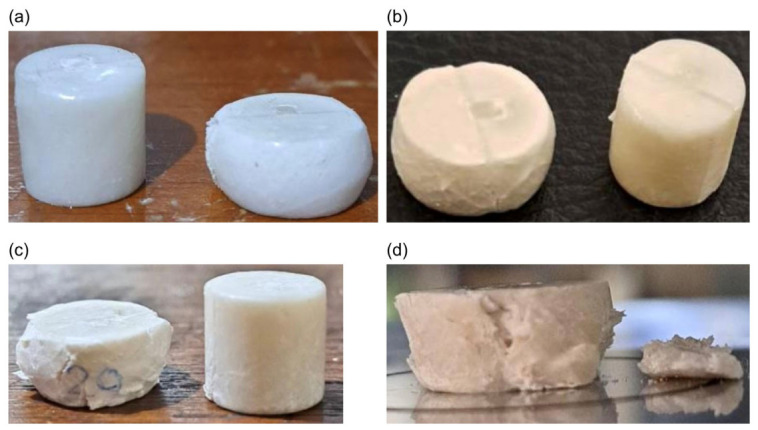
Representative photographs of the scaffolds after compressive testing. (**a**) 0%wt HA. (**b**) 10%wt HA. (**c**) 20%wt HA. (**d**) 30%wt HA.

Analysis of the load–displacement curves for the various PLA/HA specimens showed that increasing HA content progressively reduced the ultimate compressive strength of the scaffold, as the material became more brittle and less resistant to compressive loading. Up to 20% HA, the specimens still exhibited similar curve profiles, indicating that the mechanical behavior of the 0%, 10%, and 20% HA specimens remained comparable. However, at 30% HA, the curve profile changed markedly: after reaching the peak load, the specimen fractured immediately with no plastic deformation plateau, which is characteristic of brittle material failure, as shown in Figure 13.

**Figure 14:**
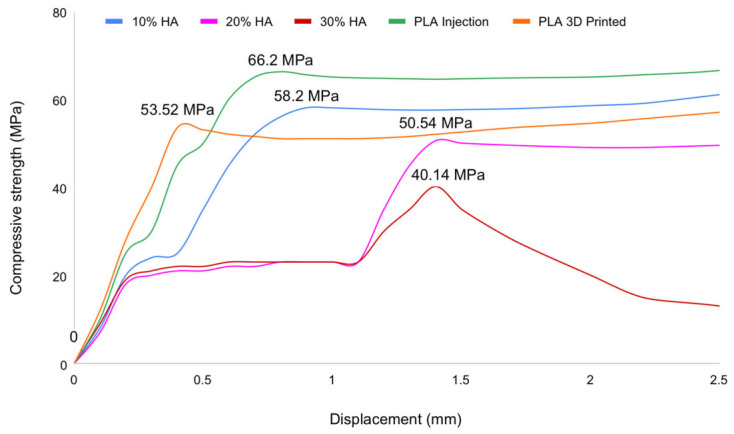
Compressive strength versus displacement curves of pure PLA and HA-reinforced composites.

Compressive testing revealed that increasing the HA proportion in PLA/HA composites leads to a progressive reduction in compressive strength (Figure 14). Injection-molded pure PLA exhibited the highest compressive strength among all compositions, while increasing HA content progressively reduced both the deformation capacity and the peak compressive stress. These findings are consistent with previous reports, which note that while HA enhances bioactivity, it possesses inherently lower mechanical strength than natural bone—particularly at high concentrations—and acts as a stress-concentrating filler phase within the polymer matrix [78]. The ultimate compressive strength for injection-molded PLA (0% HA) was 67.00 ± 0.56 MPa (compressive). With HA addition, compressive strength decreased to 56.87 ± 0.93 MPa (compressive) at 10% HA and 51.30 ± 0.98 MPa (compressive) at 20% HA. At 30% HA, the compressive strength reached its lowest value of 39.80 ± 0.87 MPa (compressive).

#### 3.3.2. Bending Testing

**Figure 15:**
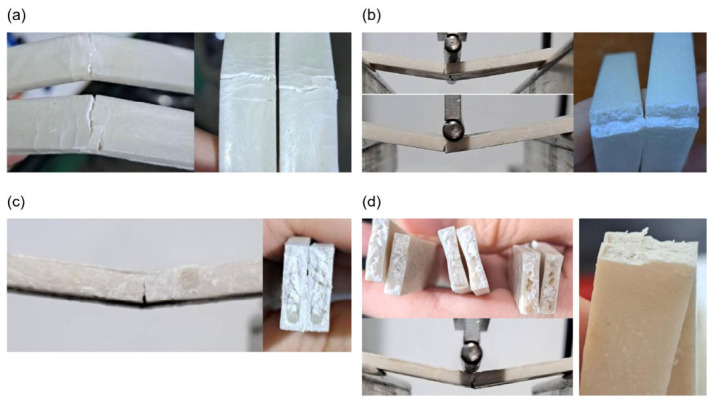
Representative photographs of the scaffolds after bending testing. (**a**) 0%wt HA. (**b**) 10%wt HA. (**c**) 20%wt HA. (**d**) 30%wt HA.

Fractographic characteristics of bending test specimens for neat PLA and PLA/HA composite scaffolds are shown in Figure 15. Neat PLA exhibits ductile-dominated behavior with relatively smooth fracture surfaces and stable crack propagation along the tensile region. Under flexural loading, crack initiation occurs at the outer tensile surface of the 10%wt HA, followed by progressive crack growth toward the neutral axis. The incorporation of 20%wt HA induces a transition toward brittle fracture, as evidenced by reduced plastic deformation and the presence of irregular, rough fracture features. At higher HA contents as presented in 30%wt HA specimen, the fracture morphology becomes increasingly heterogeneous, with pronounced micro-voids and interfacial debonding, indicating stress concentration sites and limited load transfer efficiency within the scaffold matrix.

**Figure 16:**
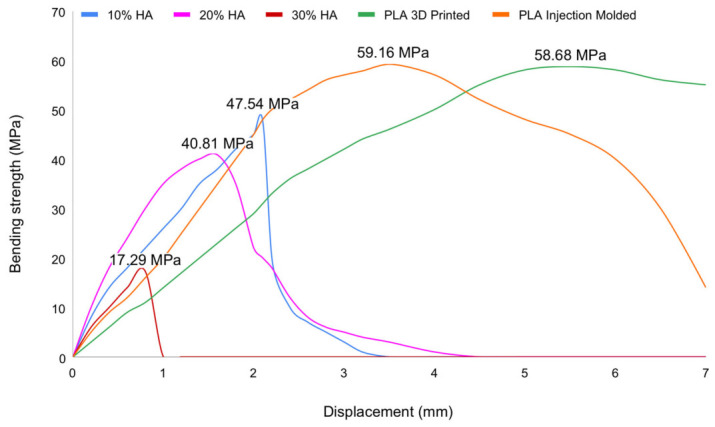
Bending strength versus displacement curves of pure PLA and HA-reinforced composites.

The bending strength–displacement relationship for each HA ratio is shown in Figure 16, and it is evident that higher HA ratios correspond to lower ultimate bending strength. A statistically significant reduction was observed between pure PLA and 10% HA specimens; however, when the HA content is increased to 20%, the reduction in bending strength is only 14.1%. A further increase in HA content beyond 20 wt% made the bending strength drops by more than 50% and the specimen becomes more brittle, with the 30% HA specimen able to withstand a displacement of less than 1 mm before failure. A comparison of injection-molded and 3D-printed pure PLA specimens showed that both fabrication methods yielded similar ultimate bending strength; however, the displacement at failure was substantially higher for 3D-printed pure PLA specimens. This phenomenon may be attributed to residual stresses in the 3D-printed pure PLA specimens, which arise from the continuous layer-by-layer heating and cooling process as well as the faster cooling rate resulting from exposure to ambient air. These residual stresses influence the plastic deformation behavior of the material, causing the specimens to undergo greater deformation prior to failure.

The bending test results demonstrate a clear negative correlation between HA content and bending strength. As HA content increases, the scaffold’s capacity to resist flexural forces diminishes. This observation is consistent with the known mechanical limitations of HA: while it provides a favorable environment for bone ingrowth, it lacks the flexibility and toughness required to resist bending stresses [21,79]. The mechanical mismatch between HA-rich scaffolds and the surrounding bone tissue may also contribute to stress concentration at the bone–implant interface, potentially leading to interfacial microfractures or implant failure under repetitive loading. In contrast to compressive loading, bending performance dropped more sharply with HA addition. The ultimate bending strength for injection-molded PLA (0% HA) was 58.60 ± 0.30 MPa (bending). With HA addition, bending strength decreased to 47.50 ± 0.75 MPa (bending) at 10% HA and 40.47 ± 0.71 MPa (bending) at 20% HA. The maximum load prior to fracture occurred at a displacement of approximately 1.5–1.8 mm. At 30% HA, the bending strength reached its lowest value of 17.07 ± 0.67 MPa (bending), and maximum load or fracture occurred at a displacement of approximately 0.80–0.85 mm.

#### 3.3.3. Tensile Testing

**Figure 17:**
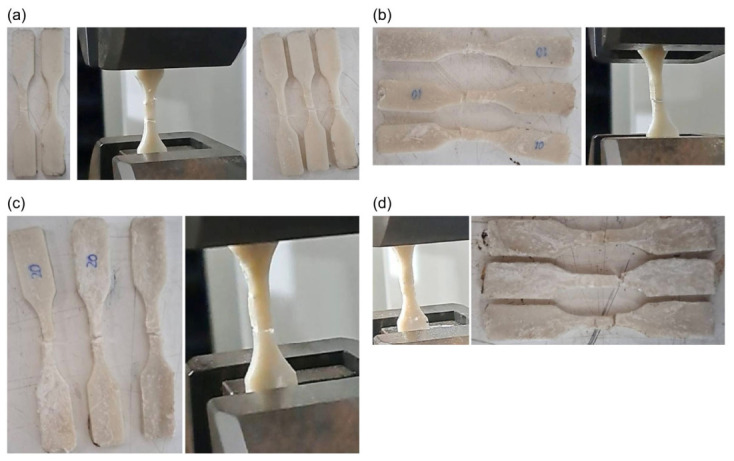
Representative photographs of the scaffolds after tensile testing. (**a**) 0%wt HA. (**b**) 10%wt HA. (**c**) 20%wt HA. (**d**) 30%wt HA.

Examination of the deformed specimens across all HA ratios confirmed that higher HA content results in more brittle specimens with greater susceptibility to fracture. Furthermore, due to the specimen geometry and the injection molding process, specimens with higher HA contents underwent dimensional distortion upon removal from the mold as a consequence of excessive brittleness. During testing, the 20% and 30% HA specimens were so brittle that they could be fractured by hand, and some specimens fractured during placement in the testing machine grips as a result of the pre-load applied. As shown in Figure 17, the addition of as little as 10% HA significantly reduced tensile strength by 43.8%, while a further 10% increase in HA content reduced tensile strength by more than 50% relative to the 10% HA specimens, rendering the material highly brittle and essentially incapable of sustaining tensile loading.

**Figure 18:**
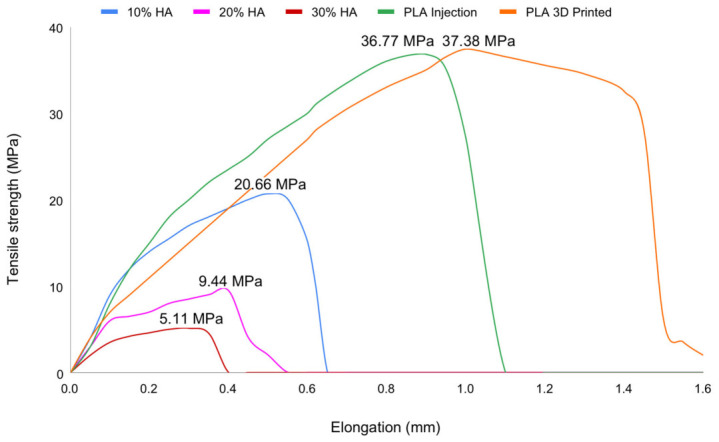
Tensile strength versus elongation curves of pure PLA and HA-reinforced composites.

Although a comparison of pure PLA specimens fabricated by 3D printing and injection molding revealed only modest differences in tensile strength, it is plausible that composites with 10%, 20%, and 30% HA would exhibit higher tensile strength if fabricated by FDM rather than injection molding. The comparison across specimens is presented in Figure 18. The ultimate tensile strength for injection-molded PLA (0% HA) was 36.70 ± 0.10 MPa (tensile). With HA addition, tensile strength decreased to 20.23 ± 0.45 MPa (tensile) at 10% HA and 9.47 ± 0.25 MPa (tensile) at 20% HA. At 30% HA, the tensile strength reached its lowest value of 5.17 ± 0.15 MPa (tensile).

The tensile test results further underscore the mechanical limitations of high-HA-content scaffolds. While HA improves bioactivity by providing osteoconductive sites and mitigating acidic degradation byproducts, its proportion must be carefully controlled to avoid an unacceptable loss of tensile performance. Based on the combined compressive, bending, and tensile data, an HA content of ≤10 wt% is recommended as the upper threshold for maintaining adequate mechanical integrity while ensuring compatibility with the load-bearing requirements of bone repair applications.

### 3.4. Mechanical Properties Evaluation

The mechanical properties of solid PLA/HA composite specimens—compressive, bending, and tensile strength—were statistically evaluated across five fabrication/composition groups using the Kruskal–Wallis test, followed by Dunn’s post hoc test with Bonferroni correction (Table 2). Non-parametric testing was selected due to the unequal group sizes and the limited number of replicates per condition, which precluded reliable assessment of normality. The number of specimens per group was not equal due to experimental constraints. Kruskal–Wallis analysis confirmed statistically significant overall differences for all three properties: compressive strength, bending strength, and tensile strength. Post hoc pairwise comparisons with Bonferroni correction were conducted across all group pairs (α = 0.05).

Table 2 summarizes the mechanical properties of PLA/HA scaffolds across HA ratio groups and shows an overall reduction in compressive, bending, and tensile strength with increasing HA content. Kruskal–Wallis test confirmed significant between-group differences for compressive strength (H = 23.077, *p* = 0.001), bending strength (H = 13.033, *p* = 0.011), and tensile strength (H = 13.233, *p* = 0.010). Among the measured properties, the tensile response exhibited the greatest sensitivity to HA loading, with the highest-HA group showing markedly lower values than the low-HA groups; Dunn’s post hoc analysis (Holm-corrected) identified a significant difference between group 1 and group 5 for tensile strength (p_adj = 0.014). A pronounced decrease was also observed in bending strength, and post hoc testing indicated that group 5 was significantly lower than group 2 (p_adj = 0.019). Compressive strength declined more gradually but remained significantly different across groups, with post hoc comparisons indicating lower compressive strength for group 5 relative to groups 2 and 3, and lower compressive strength for group 4 relative to group 2 (Holm-adjusted *p* < 0.05). Collectively, these results indicate that increasing HA content compromises load-bearing performance, consistent with reduced load transfer efficiency and the formation of microstructural stress concentrators at higher filler contents (e.g., particle agglomeration, interfacial debonding, and voids), which promote earlier crack initiation and propagation under mechanical loading. Collectively, these findings support a maximum HA content of ≤10 wt% as the practical upper limit for maintaining structural adequacy in non-load-bearing bone scaffold applications.

Several sources of experimental uncertainty are acknowledged. For FDM 3D-printed specimens, variability in layer adhesion, filament diameter, and thermal history between print runs introduces inter-specimen differences in local porosity and density, which are reflected in the reported standard deviations. For injection-molded PLA/HA specimens, manual actuation of the injection process introduces variability in injection pressure and HA particle distribution, contributing to the non-uniform surface texture and color observed at higher HA loadings. Dimensional tolerances from machining operations (bench and Struers grinding) affect cross-sectional area calculations and hence derived stress values. Machine measurement uncertainty is estimated at ±2% of the measured load, based on equipment calibration records. Given the small sample size (*n* = 3–5 per group), the results should be interpreted as a preliminary screening study.

#### 3.4.1. Effect of Fabrication Method on Pure PLA Mechanical Properties

Comparison of the two pure PLA groups revealed that the fabrication method had a loading-mode-dependent effect on mechanical performance. Injection-molded pure PLA (PLA IM: 67.00 ± 0.56 MPa) exhibited compressive strength 23.2% higher than FDM-printed pure PLA (PLA FDM: 54.40 ± 0.64 MPa); however, this difference did not reach statistical significance after Bonferroni correction (*p* = 0.577), reflecting the conservative nature of the correction at small sample sizes. In contrast, bending strength was virtually identical between fabrication methods (PLA FDM: 58.40 ± 0.68 MPa; PLA IM: 58.60 ± 0.30 MPa; *p* = 1.000), as was tensile strength (PLA FDM: 37.54 ± 0.40 MPa; PLA IM: 36.70 ± 0.10 MPa; *p* = 1.000). These findings indicate that for bending and tensile loading modes, FDM and injection molding produce mechanically equivalent pure PLA material, consistent with published reports that FDM specimens achieve bulk polymer tensile and flexural properties under optimized printing parameters [80]. Conclusions about geometric superiority in isolation cannot be drawn from the present study.

#### 3.4.2. Effect of HA Content on Compressive Strength

Compressive strength monotonically declined with increasing HA content, from 67.00 ± 0.56 MPa (0 wt%) to 56.87 ± 0.93 MPa (10 wt%), 51.30 ± 0.98 MPa (20 wt%), and 39.80 ± 0.87 MPa (30 wt%), corresponding to an overall reduction of 40.6%. The Kruskal–Wallis test confirmed significant differences across groups (*p* = 0.001), consistent with Table 2. Post hoc analysis indicated that the 30 wt% HA group was significantly lower than the 10 wt% and 20 wt% groups, while the 20 wt% group also showed reduced strength relative to 10 wt% HA. Despite limited pairwise significance due to conservative correction, the progressive decline supports a dose-dependent deterioration in compressive performance. This behavior is attributed to HA-induced microstructural heterogeneity, including particle agglomeration and interfacial debonding, which act as stress concentrators. Nevertheless, all groups retained compressive strength well above the trabecular bone range, indicating suitability for non-load-bearing applications.

The absence of significant pairwise differences at intermediate HA concentrations reflects the conservative Bonferroni correction combined with small group sizes, rather than an absence of a true progressive effect—the monotonic decline in means across groups is biologically consistent and supports the interpretation of a dose-dependent reduction in compressive integrity. HA particles act as stress-concentrating filler inclusions within the PLA matrix; at elevated loading fractions, particle agglomeration generates localized void clusters that accelerate compressive failure by promoting internal buckling and shear band formation [81,82]. Importantly, even the 30% HA compressive mean (39.80 MPa) substantially exceeds the upper bound of trabecular bone compressive strength (1.5–7.8 MPa), indicating that all compositions tested retain sufficient compressive capacity for non-load-bearing cancellous bone defect applications [83].

#### 3.4.3. Effect of HA Content on Bending Strength

Bending strength exhibited a more pronounced reduction than compressive strength, decreasing from 58.60 ± 0.30 MPa (0 wt%) to 47.50 ± 0.75 MPa (10 wt%), 40.47 ± 0.71 MPa (20 wt%), and 17.07 ± 0.67 MPa (30 wt%), representing a 70.9% decrease at the highest HA content. Statistical analysis confirmed significant differences between groups (*p* = 0.011), with post hoc testing identifying the 30 wt% HA group as significantly lower than at least one lower-HA group (Table 2).

The sharp decline at 30 wt% HA indicates a transition from ductile to brittle behavior, consistent with premature fracture under flexural loading. This is attributed to increased defect density and weak particle–matrix interfaces, which accelerate crack initiation and propagation under tensile stresses during bending. While ≤20 wt% HA maintains moderate bending capacity, the 30 wt% condition falls below mechanically acceptable limits for structural scaffold applications.

The disproportionately large reduction at 30 wt% HA—exceeding 70% relative to both pure PLA baselines—is consistent with the brittle fracture morphology observed in that group, where specimens failed at deflections below 1 mm with no discernible plastic plateau. This abrupt transition reflects the role of HA agglomeration at high loading fractions in creating a dense population of interfacial debonding sites that rapidly coalesce under the tensile surface stresses generated during three-point bending, resulting in catastrophic crack propagation rather than progressive yielding [82,84]. The bending strength of all groups containing ≤20% HA (40.47–58.60 MPa) remains within a range considered mechanically adequate for bone scaffold applications, whereas the 30% HA group falls to a level that would be clinically inadequate for any load-transmitting bone defect site.

#### 3.4.4. Effect of HA Content on Tensile Strength

Tensile strength exhibited the steepest and most clinically consequential decline with increasing HA content. Tensile strength showed the highest sensitivity to HA addition, decreasing from 36.70 ± 0.10 MPa (0 wt%) to 20.23 ± 0.45 MPa (10 wt%), 9.47 ± 0.25 MPa (20 wt%), and 5.17 ± 0.15 MPa (30 wt%), corresponding to an 86.2% reduction. The Kruskal–Wallis test confirmed significant differences among groups (*p* = 0.010), with post hoc analysis identifying a significant difference between the lowest and highest HA contents (Table 2). The marked degradation in tensile performance reflects poor stress transfer efficiency and increased brittleness at higher HA loadings. Severe particle agglomeration and void formation promote early crack initiation, and specimens at ≥20 wt% HA exhibited brittle fracture behavior, in some cases failing during handling. These findings indicate that tensile load-bearing capacity becomes negligible at higher HA concentrations, limiting their applicability in mechanically demanding environments.

Many PLA/HA systems show an initial increase in tensile strength at low HA (≈2.5–10 wt%), followed by a steep drop at higher HA due to particle agglomeration, increased porosity, and poor stress transfer [81,82]. During testing, 20% and 30% HA specimens were so brittle that some fractured during grip placement, precluding reliable measurement and confirming that tensile load-bearing capacity at these HA levels is effectively negligible for physiological applications.

#### 3.4.5. Integrated Interpretation and Clinical Implications

The combined mechanical results demonstrate a consistent inverse relationship between HA content and structural performance across compressive, bending, and tensile modes (Table 2). While compressive strength decreased moderately with increasing HA content, bending and tensile properties exhibited substantially greater sensitivity, particularly at ≥20 wt% HA. This divergence reflects the increasing dominance of tensile-driven failure mechanisms, where interfacial defects and particle agglomeration critically impair stress transfer.

All mechanical parameters showed significant differences across HA contents (*p* < 0.05), although post hoc analysis indicated that the most pronounced reductions were associated with the 30 wt% HA group. This suggests the presence of a threshold beyond which mechanical degradation becomes disproportionately severe. In contrast, the 10 wt% HA composition maintained comparatively higher strength values across all loading conditions, with only moderate reductions relative to the control.

From a materials design perspective, these findings indicate that low HA incorporation (≤10 wt%) provides a balanced compromise between mechanical integrity and bioactive reinforcement. Higher HA contents, while potentially beneficial for biological performance, introduce structural heterogeneities that compromise load-bearing capacity. Therefore, the 10 wt% HA formulation is identified as the optimal composition within the investigated range for applications requiring both mechanical reliability and biofunctional enhancement.

### 3.5. Lattice Structures in 3D-Printed Bone Applications

All four geometries are readily printable via FDM using standard slicer software (PrusaSlicer), confirming manufacturing feasibility. FDM and similar extrusion-based method can produce porous scaffolds with porosity errors below 5% in well-controlled conditions, indicating relatively high accuracy of pore structure which make it suitable to construct precise lattice and TPMS-based architectures for bone scaffolds [85]. The scaffold designs evaluated are mechanically suited for bone scaffold engineering by virtue of their capacity to sustain high compressive and bending loads, which makes it more load-bearing than a cancellous-bone-specific match. This study is relevant to large bone defect repair in general, but not all tested dimensions and mechanical ranges are specifically optimized for cancellous bone repair.

Previous studies have demonstrated that gyroid TPMS scaffolds fabricated via FDM exhibit mechanical properties comparable to those of cortical bone, establishing their suitability as candidate structures for bone substitution and orthopaedic implant applications [86,87]. However, mechanical performance and geometric fidelity are strongly dependent on print orientation as well as other process parameters. Thus, careful print orientation selection, to manage overhang-like regions and anisotropy, and parameter optimization are essential when using gyroid TPMS in FDM [88].

The parametric nature of all four infill patterns allows straightforward scaling to patient-specific scaffold volumes by adjusting the bounding geometry in AutoCAD 2023 (Autodesk) software without changing the infill parameters, making all four designs clinically scalable. All tested geometries in this study were evaluated under quasi-static loading only. The reported compression rate of 1 mm/min and bending crosshead rate of 0.01 mm/s are consistent with quasi-static mechanical testing. Assessment of FDM-printed bone scaffold lattices under cyclic loading conditions is essential for clinical translation. Multiple FDM and related 3D-printed bone-scaffold lattices must be tested under cyclic loading. Fatigue performance depends strongly on geometry, porosity, printing parameters, and several architectures demonstrate adequate or even robust fatigue resistance within physiologic cancellous-bone stress ranges [89]. Fatigue performance was not assessed in this study and must be characterized before clinical translation for future work.

## 4. Conclusions

### 4.1. General Conclusions

This study systematically investigated the mechanical performance of FDM-printed PLA scaffolds incorporating four infill geometries—rectangular, triangular, gyroid, and 3D honeycomb—alongside injection-molded PLA/HA composites with HA contents ranging from 0 to 30 wt%, under compressive, flexural, and tensile loading conditions. The principal findings are threefold. First, the rectangular infill pattern at 70% infill density yielded the most favorable load-to-weight ratio and the greatest plastic deformation capacity prior to fracture among the four geometries evaluated, identifying it as a mechanically suitable candidate for large bone defect repair in non-primary load-bearing sites. It should be noted that this advantage reflects the combined effect of both geometry and infill density, as rectangular and triangular patterns were tested at 70% density while gyroid and 3D honeycomb were tested at 40% density to achieve comparable scaffold porosity. Second, HA incorporation beyond 10 wt% substantially compromised mechanical strength across all loading modes—with tensile strength declining by 46.1% at 10 wt% HA and 86.2% at 30 wt% HA—indicating that HA addition should be limited to ≤10 wt% to preserve adequate structural integrity. Third, infill density was identified as the dominant determinant of macroscale mechanical performance at the porosity levels examined, with infill geometry exerting a comparatively secondary influence. Collectively, these findings provide a rational basis for the design of 3D-printed PLA/HA scaffolds for bone defect repair, and serve as a foundation for future studies incorporating fatigue testing, microstructural characterization, and biological validation.

### 4.2. Directions for Future Work

The present study is subject to several limitations that should be acknowledged. The use of distinct manufacturing methods for pure PLA and PLA/HA specimens introduces a confounding variable that precludes direct cross-method comparison of absolute mechanical values. Mechanical evaluation was confined to quasi-static loading conditions, and neither fatigue nor dynamic performance was assessed. Furthermore, FE analysis was not conducted, and microstructural characterization techniques, including SEM and micro-computed tomography (micro-CT), were outside the scope of this work. The sample size of *n* ≤ 5 per group was deemed adequate for preliminary screening purposes; however, it is insufficient to support definitive statistical conclusions. In addition, the sharp-edge junctions inherent to the rectangular and triangular FDM infill geometries introduce stress concentrations that were not quantified in the current investigation. Finally, all biological assertions presented herein are derived from the existing literature and necessitate experimental validation prior to broader application.

Building upon these limitations, several directions are proposed for future investigation. FE analysis of all four scaffold geometries should be prioritized to quantify junction stress concentrations and elucidate internal stress distributions. The application of TPMS-based junction-smoothing strategies to the rectangular and triangular infill designs is recommended to enhance fatigue resistance. Subsequent studies should incorporate fatigue testing under physiologically relevant cyclic loading conditions, alongside SEM and micro-CT characterization of HA distribution and scaffold microarchitecture. In vitro and in vivo biological evaluations are essential to substantiate the osteogenic potential suggested by the reviewed literature. Moreover, systematic optimization of HA loading within the 0–10 wt% range warrants dedicated investigation. Collectively, these directions represent critical advancements that extend beyond the scope of the present study and are identified as essential steps toward the development of clinically translatable scaffolds.

## Figures and Tables

**Table 1:** Default printing parameters.

Parameter	Specification
Nozzle diameter	0.4 mm
Printing speed	60 mm/s
Bed temperature (PLA)	60 °C
Nozzle temperature (PLA)	215 °C
Layer thickness	0.3 mm
First layer height	0.2 mm
Shell thickness	0 perimeters
Bottom thickness	0.5 mm (3 layers)
Top thickness	0.7 mm (4 layers)

**Table 2:** Mechanical properties of scaffolds across HA-reinforced series.

HA Ratio	Compressive Strength (MPa)	Bending Strength (MPa)	Tensile Strength (MPa)
	*n* = 5	*n* = 3	*n* = 3
FDM 3D-printed PLA (0% HA)	54.37 ± 0.69 ^ab^	57.44 ± 0.84 ^ab^	37.19 ± 0.55 ^a^
Injection-molded PLA (0% HA)	66.44 ± 0.70 ^a^	58.20 ± 0.39 ^a^	36.30 ± 0.38 ^ab^
PLA/HA (10% HA)	56.94 ± 1.08 ^a^	47.21 ± 0.29 ^ab^	20.26 ± 0.34 ^ab^
PLA/HA (20% HA)	51.00 ± 0.30 ^b^	40.16 ± 0.36 ^ab^	9.29 ± 0.17 ^ab^
PLA/HA (30% HA)	39.50 ± 0.49 ^b^	16.95 ± 0.41 ^b^	5.17 ± 0.18 ^b^
Kruskal–Wallis H (*p*)	23.077 (*p* = 0.001)	13.033 (*p* = 0.011)	13.233 (*p* = 0.010)

Values are presented as mean ± SD. Different superscript letters within the same column indicate significant differences between HA ratio groups based on Dunn’s post hoc test with Holm correction (*p* < 0.05). Groups sharing at least one letter are not significantly different.

## Data Availability

The original contributions presented in this study are included in the article. Further inquiries can be directed to the corresponding authors.

## Abbreviations

The following abbreviations are used in this manuscript:

PLA	Polylactic acid
HA	Hydroxyapatite
n-HA	Nano-hydroxyapatite
FDM	Fused-deposition modeling
3D	Three-dimensional
TPMS	Triply periodic minimal surface
AM	Additive manufacturing
FE	Finite element
SD	Standard deviation
SEM	Scanning electron microscopy
